# Impact of antimalarial resistance and COVID-19 pandemic on malaria care among pregnant women in Northern Uganda (ERASE): protocol of a prospective observational study

**DOI:** 10.1186/s12879-022-07645-3

**Published:** 2022-08-04

**Authors:** Francesco Vladimiro Segala, Francesco Di Gennaro, Jerry Ictho, Mariangela L’Episcopia, Emmanuel Onapa, Claudia Marotta, Elda De Vita, James Amone, Valentina Iacobelli, Joseph Ogwang, Giovanni Dall’Oglio, Benedict Ngole, Rita Murri, Lameck Olal, Massimo Fantoni, Samuel Okori, Giovanni Putoto, Carlo Severini, Peter Lochoro, Annalisa Saracino

**Affiliations:** 1 Dipartimento di Scienze di Laboratorio e Infettivologiche, Fondazione Policlinico Universitario “A. Gemelli” IRCCS, Rome, Italy; 2 Department of Biomedical Sciences and Human Oncology, Clinic of Infectious Diseases, University of Bari, Bari, Italy; 3 Doctors with Africa, CUAMM, Kampala, Uganda; 4 Department of Infectious Diseases, Istituto Superiore di Sanità, Rome, Italy; 5 St. John’s XXIII Hospital Aber, Jaber, Uganda; 6 Doctors with Africa Cuamm, Padua, Italy; 7 Department Woman and Child Health Sciences, Catholic University of the Sacred Heart, Rome, Italy; 8 African Network for Change, Kampala, Uganda; 9 Dipartimento di Sicurezza e Bioetica-Sezione di Malattie Infettive, Università Cattolica del Sacro Cuore, Rome, Italy

**Keywords:** Malaria in pregnancy, COVID-19, Antimalarial resistance, Sulfadoxine-pyrimethamine, Artemisinin derivatives

## Abstract

**Background:**

Uganda accounts for 5% of all malaria cases and deaths reported globally and, in endemic countries, pregnancy is a risk factor for both acquisition of *P. falciparum* infection and development of severe malaria. In recent years, malaria control has been threatened by COVID-19 pandemic and by the emergence, in Northern Uganda, of both resistance to artemisinin derivatives and to sulfadoxine-pyrimethamine.

**Methods:**

In this facility-based, prospective, observational study, pregnant women will be recruited at antenatal-care visits and followed-up until delivery. Collected data will explore the incidence of asymptomatic parasitemia and malaria-related outcomes, as well as the attitudes towards malaria prevention, administration of intermittent preventive treatment, healthcare seeking behavior and use of insecticide-treated nets. A subpopulation of women diagnosed with malaria will be recruited and their blood samples will be analyzed for detection of genetic markers of resistance to artemisinin derivatives and sulfadoxine-pyrimethamine. Also, to investigate the impact of COVID-19 on malaria care among pregnant women, a retrospective, interrupted-time series will be conducted on at the study sites for the period January 2018 to December 2021.

**Discussion:**

The present study will explore the impact of COVID-19 pandemic on incidence of malaria and malaria-related adverse outcomes, along with the prevalence of resistance to artemisinin derivatives and to sulfadoxine-pyrimethamine. To our knowledge, this is the first study aiming to explore the combined effect of these factors on a cohort of pregnant women.

*Trial registration*: This study has been registered on the ClinicalTrials.gov public website on 26th April, 2022. ClinicalTrials.gov Identifier: NCT05348746.

## Background

### Malaria epidemiology and COVID-19

Over the last twenty years, tremendous progress has been made on malaria control, averting an estimate of 1.5 billion cases and saving 7.6 million lives. However, despite the astonishing results achieved in the last two decades, COVID-19 pandemic added a crucial challenge to the fight against the disease. On one side, malaria control relies heavily on individual choice to seek care, and early messaging targeted on reducing SARS-CoV2 transmission advised people to stay home in case of fever. On the other side, supply chains that allow the delivery of malaria commodities, such as insecticide-treated nets or antimalarial drugs, have been disrupted, and healthcare workforce constrained. All these factors contributed to a reverse of the reducing trend in malaria cases and deaths that shifted, respectively, from 227 million and 534,000 in 2019 to 241 million and 602,000 in 2020 [1].

According to the latest WHO World Malaria Report [1], Uganda accounts for 5% of all malaria cases reported globally. Oyam and Kole districts, selected for the study, are among the most affected areas in the country with, respectively, 407 and 361 new cases per 1000 inhabitants in 2019 [2]. In this context, particularly affected are children under five years of age (U5) and pregnant women. In the area where the project will operate, the rate of pregnant women that receives 3 or more doses of intermittent preventive treatment (IPTp) is less than 48% [2].

Concerning the impact of COVID-19 on malaria care in Uganda, a study conducted by Namuganga et al. [3]—except for a modest decrease in the proportion of malaria cases treated with ACT—documented no major effects on malaria disease burden. However, the study did not evaluate the impact of COVID-19 on antenatal care and malaria-related maternal outcomes. At this regard, in the pre-pandemic period (years 2018–2019) the mean antenatal care (ANC) attendance in the district of Oyam was of 1721 first visits per month, while an average of 472 women per month received at least 3 doses of IPTp.

### Pregnancy associated malaria

#### Intermittent preventive treatment

In hyperendemic areas, pregnancy is a risk factor for both acquisition [4] of *P. falciparum* infection and for development of severe malaria [5]. Younger women, primi- or secundigravidae and HIV + women are particularly at risk [6]. Adverse outcomes for mothers and their infants include maternal anemia, low birthweight, prematurity, placental malaria, infant malaria, infant anemia and congenital malaria. Furthermore, adverse events are exacerbated by poor maternal nutritional status [7] and HIV coinfection [8].

Apart from consistent use of ITN, malaria control in this population is based on two pillars: intermittent preventive treatment of asymptomatic women and appropriate management in case of illness [9]. IPTp with sulfadoxine-pyrimethamine (SP) is still highly cost-effective in preventing the adverse consequences of malaria on maternal and foetal outcomes, even in areas with a high prevalence of quintuple mutant parasites [10], but coverage remains unacceptably low in several African countries [11]. For HIV-negative pregnant women, IPTp consists in the administration of at least three doses of SP (1500 mg sulfadoxine/75 mg pyrimethamine), in three antenatal care visits, starting early in the second trimester and at least four weeks apart [9].

In HIV negative women, a promising alternative to SP for IPTp is dihydroartemisinin-piperaquine (DHA-PPQ, 3 full strength tabs, 40 mg/320 mg, given once a day for 3 consecutive days), that showed to be more efficacious in reducing maternal malaria parasitemia and anemia at delivery, stillbirths and early infant mortality. In fact, SP efficacy may be decreased in areas with very high drug resistance and consistent presence of sextuple mutant haplotypes of *P. falciparum* [12]. However, there is no consensus as to the level of resistance at which SP-IPTp should be discontinued and an alternative regimen substituted.

For HIV positive patients, the current WHO guidelines recommend daily co-trimossazole prophylaxis.

#### Diagnosis and treatment

In most endemic countries, diagnosis heavily relies on the use of rapid diagnostic tests (RDT) which, however, are insufficiently sensitive in detecting the so-called subpatent infections—asymptomatic infections with low parasite densities—and infections due to parasites carrying the pfhrp2 and pfhrp3 gene deletions [13]. These limitations are partly overcome by microscopy and PCR-based tests, that can detect also low parasitemia [14], but the clinical impact of such infections is still matter of debate [15].

Treatment of malaria differs according to gestational age. For women in their first trimester with uncomplicated *P. falciparum* malaria, WHO recommends 7 days of quinine + clindamycin. From the second trimester on, experience with artemisinin derivatives is increasingly reassuring: no adverse effects on the mother or foetus have been reported. Thus, treatment of uncomplicated *P. falciparum* malaria consists of three-day course of oral artemisinin-based combination therapy (ACT). On the other side, treatment of severe malaria does not differ from the one prescribed to non-pregnant women. Parenteral artesunate is the treatment of choice in all trimesters.

### Resistance to sulfadoxine-pyrimethamine and artemisinin derivatives

Another substantial challenge for malaria case-management is resistance to first line drugs, namely artesunate and ACT. A recent paper published by Balikagala et al. documented, for the first time in African history, the presence of artemisinin resistance in a longitudinal study conducted in Gulu, Uganda [16]. According to this study, single-nucleotide polymorphisms haplotypes associated with artemisinin resistance (i.e., mutations involving the gene locus *kelch13*) clearly showed the substantial difference of haplotypes between A675V isolates in Uganda and in Southeast Asia, which suggested that the mutation probably emerged independently in Africa and Southeast Asia. In Africa, potential factors that may contribute to a delayed emergence and spread of artemisinin resistance are the extent of acquired immunity, the rate of polyclonal infections and of chronic asymptomatic infections [17]. However, the constant selective pressure exerted by the widespread use of ACT pose a substantial threat for the emergence of clinically relevant forms of resistance.

**Fig. 1:**
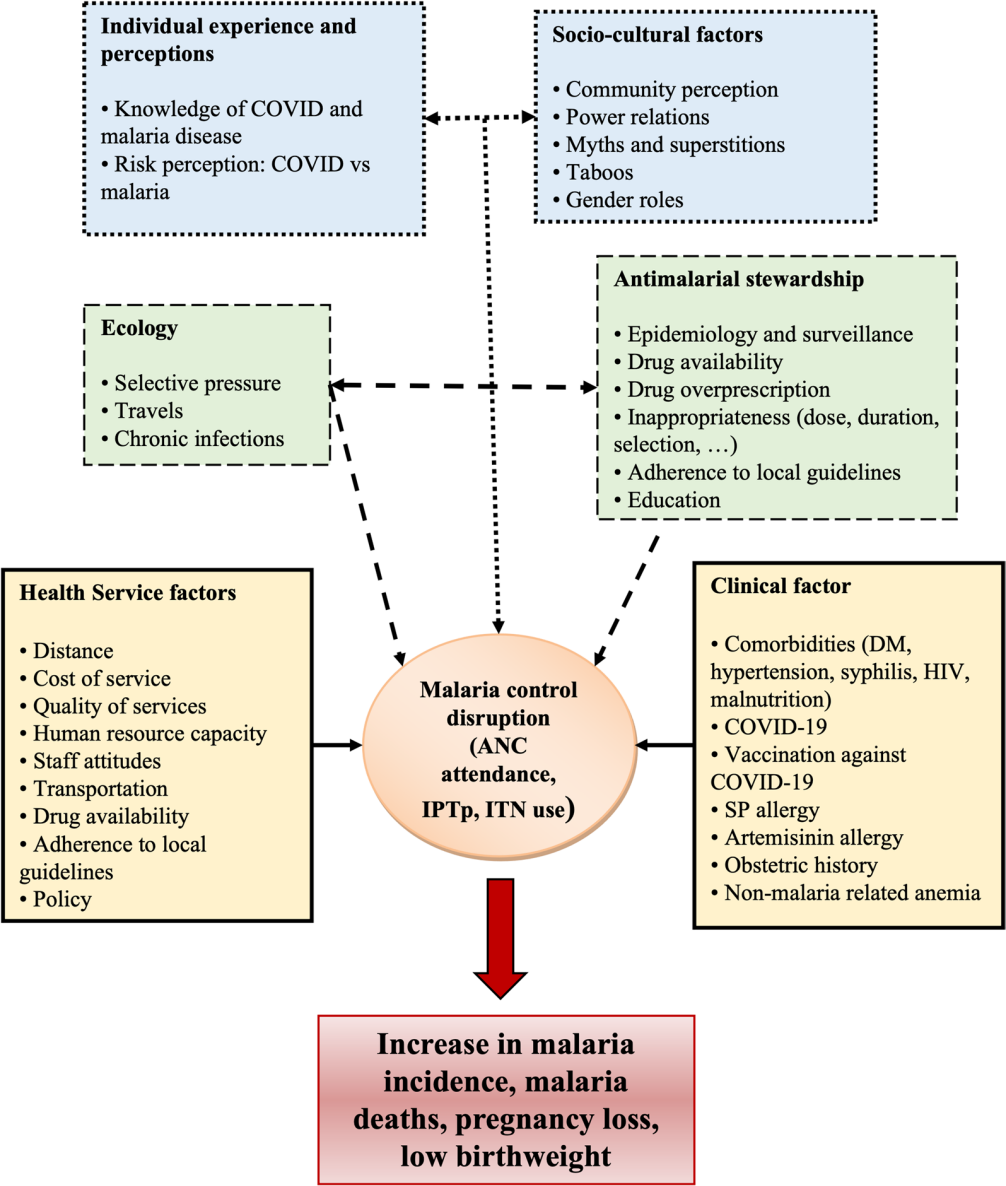
Conceptual framework showing the possible impact of COVID-19 and antimalarial resistance on malaria care among pregnant women. Dashed line (green): antimalarial resistance; Dotted line (blue): COVID-19; Continuous line (yellow): both COVID-19 and antimalarial resistance

Furthermore, a study conducted by Mbonye et al. [18] in 2015 documented a baseline prevalence of *Pfdhfr* and *Pfdhps* mutations—conferring resistance to SP in *P. falciparum*—to be 89% for the quintuple mutated haplotype and 3.9% for the sextuple mutated haplotype, reaching 16.7% after one dose of SP. Today’s prevalence of the sextuple mutated haplotypes, potentially impairing the effectiveness of SP-IPTp is not known. Conceptual framework of the study is provided in Fig. 1.

## Methods

### Study design

This will be a facility-based, prospective observational study, using quantitative methods of data collection. Semi-structured questionnaires will be administered to collect the data. The study will be conducted on three separate populations, that is: “Cohort of pregnant women”, “Antimalarial resistance sub-population”, and “COVID-19 impact population” for which study methods are described separately.

#### Cohort of pregnant women

The data will be collected following a cohort of pregnant women presenting to antenatal care visits. We shall have both a retrospective cohort for the period January 2018 to December 2021 to determine the Impact of COVID-19 pandemic on malaria control and a prospective cohort for the period July 2022 to June 2024 to determine the incidence of malaria related adverse maternal and foetal outcomes. For the prospective cohort, recruitment will take place at ANC clinic. Collected data will explore the practices towards malaria prevention during the COVID-19 pandemic, malaria and COVID risk-perception and use of insecticide-treated nets, while and follow up will investigate access to antenatal care visits, administration of IPTp, healthcare seeking behaviour in case of fever. Follow-up will end at delivery, when maternal and foetal outcomes will be collected.

#### Antimalarial resistance population

To estimate the epidemiological burden of resistance to first-line drugs for treatment and prevention of malaria in pregnant women, a separate sub-population of women diagnosed with positive malaria parasitaemia will be recruited. Prevalence of antimalaria resistance will be evaluated with a cross-sectional analysis of genetic polymorphisms in plasmodium parasites isolated from blood samples collected for the period July 2022 to June 2024. Individuals eligible for this subpopulation will be all pregnant women presenting with microscopically confirmed *P. falciparum* malaria in the study sites. For individuals included in this population, clinical data will be collected, and blood samples to be sent to Italian National Institute of Health, Rome, for detection of genetic markers of resistance to artemisinin derivatives and sulfadoxine-pyrimethamine.

#### COVID-19 impact population

To investigate the impact of COVID-19 pandemic on malaria care, we will conduct a retrospective time-interrupted series. Data will be collected at multiple and equally spaced time points (monthly) comparing trends in two different time periods: “pre-pandemic”, from January 2018 to December 2019, and “during pandemic”, from January 2020 to December 2021. Facility-based aggregate data will be extracted about the following indicators: total admissions in maternity ward, total deliveries in maternity unit, women presenting to first ANC contact, women presenting to fourth ANC contact, administration of at least one dose of IPTp, administration of at least three doses of IPTp, number of pregnant women presenting to outpatient visits, number of pregnant women diagnosed of malaria during outpatient clinic, number of pregnant women diagnosed of severe malaria, total number of stillbirths.

### Eligibility criteria

#### Cohort of pregnant women

Inclusion criteria:

All pregnant women at any gestational age presenting to the study sites, both at the emergency department, outpatient or ANC clinic will be eligible to participate in this study. Inclusion criteria will be:

- Pregnancy confirmed by obstetrical visit, positive urine pregnancy test or intrauterine pregnancy by ultrasound
- Provision of informed consent
- Plan to deliver in the hospital

Exclusion criteria:

- Ongoing labor or delivery

#### Antimalarial resistance population

All pregnant women presenting to Aber Hospital and selected healthcare facilities with microbiologically confirmed malaria will be eligible for recruitment.

Inclusion criteria:

- Microscopically-confirmed diagnosis of malaria
- Pregnancy confirmed by obstetrical visit, positive urine pregnancy test or intrauterine pregnancy by ultrasound
- Provision of informed consent
- Agreement to avoid antimalarial medications given outside the Uganda Clinical Guidelines [19]

Exclusion criteria:

- Too ill to participate in the study according to clinician judgment
- Intake of any antimalarial medication before recruitment

### Sample size estimation

#### Sample size estimation

- COVID-19 impact population

For the retrospective cohort investigating the impact of COVID-19, we shall use a *facility-based census* to include all pregnant mothers who sought care at the study sites for the period January 2018 to December 2021.

- Prospective çohort of pregnant women

For the prospective cohort, the sample size was estimated using the sample size estimation function in STATA12 for two-sample comparison of proportions. Null hypothesis: p1 = p2, (no difference in preterm birth rates), where p1 is the preterm birth rate among pregnant mothers diagnosed with malaria (exposed group) and p2 is the preterm birth rate among pregnant mothers with no diagnosis of malaria (non-exposed group). Assuming a type I error, alpha, of 0.050 for two-sided hypothesis, power of the study at 0.80, p1 = 0.075 and p2 = 0.039 based on the preterm birth rates reported in the Uganda Birth Cohort Study conducted from 2014–2016 in 12 districts in rural northern and southwestern Uganda [20]. Assuming equal number of participants in both groups, the required sample sizes in each of the two groups is 705. And after factoring in 10% for non-response in both groups, the total minimum required sample size is 1552 pregnant women (776 in each of the 2 groups).

- Antimalarial resistance population

Given the total population of 779,600 in Oyam and Kole districts. Given that, based on Uganda bureau of statistics (UBOS), the total number of pregnant women in the two districts is expected to be 5% of the total population (n = 38,980); the expected rate of parasitemia among pregnant mother is expected to be 27% (n = 10,525) [21]; the expected resistance to sulfadoxine-pirimethamine and artemisinin-derivatives are, respectively, 16% and 20% [16, 18]; using the sample size calculation formula developed by Daniel and colleagues [22] and a margin of error, alpha = 5% the minimum required number of pregnant women diagnosed with malaria is 203. Applying 10% correction factor and assuming an increasing trend the required minimum sample size for this population is 224 cases of microscopically confirmed malaria among pregnant women.

### Sampling procedure and selection of participants

Sampling of health Units: The study will use purposive sampling to include Aber Hospital, Aboke HCIV and Atipe HCIII as the site for enrollment. This is based on the following criteria:

- Already existent good laboratory capacity for diagnosis of malaria.
- Presence of an experienced microscopist.
- Antenatal care visit volumes of at least 50 visits per month.
- Good quality of routine service offered to patients with malaria as per periodic quality of care assessment.

Participants coming to the sampled in healthcare facilities that will meet the eligibility criteria will be included in the study. Consecutive enrollment of participants will be undertaken up to when the minimum sample size required for the study will be met.

### Study procedures

#### Microscopy

Thick and thin blood smears will be stained with 2% Giemsa and read by experienced laboratory technologists. Parasite densities will be calculated by counting the number of asexual parasites per 200 leukocytes (or per 500 leukocytes, if the count is < 10 asexual parasites/200 leukocytes), assuming a leukocyte count of 8000/µl. A blood smear will be considered negative when the examination of 100 high power fields does not reveal asexual parasites. Gametocytemia will also be determined from thick smears. Thin smears will be used for parasite species identification. At the time of delivery, recruited women will be screened for parasitemia on placental blood. This will be done by microscopy, with the same methods described above.

#### Molecular diagnosis and Plasmodium species confirmation

The blood samples of the patients will be collected using filter paper (Whatman 3 MM) during admission to the healthcare facility. The dried blood spots (DBSs) will be collected through a finger prick (three drops of blood per participant) on filter papers which will be dried and kept in plastic bags with desiccant and stored in boxes in a cool dry place at room temperature before being transferred at the ISS for molecular diagnosis and drug resistance analysis.

#### Shipment of the blood samples

The collected blood samples will be shipped to Italy for advanced polymorphism analysis. During shipment, all the samples will be stored in a dry, cool place at room temperature to the Italian National Institute of Health (Istituto Superiore di Sanità, ISS). To enhance local capacity building, one laboratory person from Aber hospital will attend a two weeks exposure at the reference laboratory in Italy.

#### Advanced analysis of polymorphism

Total genomic DNA will be extracted from filter blots (3MM Whatman) using the PureLink Genomic DNA Kits-Invitrogen, according to the manufacturer’s recommendation. Parasite identification is based on nested PCR assay targeting the 18S rRNA gene [23]. The 18S rRNA gene is used as a target since it contains both highly conserved and variable regions for each *Plasmodium* species. The genus-specific PCR will be followed by *Plasmodium* species-specific PCR amplification. Amplicons from the second PCR will be separated by electrophoresis on a 2% agarose gel and stained with ethidium bromide for visualization using ultraviolet trans-illumination. The presence of parasitaemia will be confirmed when the expected band size corresponding to *P. falciparum*, *P. vivax, P. malariae and or P. ovale* will be identified.

#### Assessment of Plasmodium falciparum drug resistance.

Target *P. falciparum* drug resistance genes: *Pfk13* propeller, *Pfdhfr* and *Pfdhps*.

The polymorphisms analysis of the propeller domain of the *Pfk13* gene will be performed by PCR amplifications and subsequent sequencing. Analysis of *Pfdhfr* gene at codons 51, 59, 108 and *Pfdhps* gene at codon positions 436, 437, 540, 581, 613 will be done by means of amplifications and subsequent Sanger sequencing. Commercial oligonucleotide primer pairs for *Pfk13* will be obtained based on the published article by Taylor et al. [24], whereas for the analysis of *dhfr* and *dhps* genes primer pairs will be obtained based on the published article by Menegon et al. [25]. The obtained sequences will be compiled and analyzed by Accelrys DS Gene software. PlasmoDB gene identification no. PF3D7_1343700 (*P. falciparum* 3D7 strain) will be used as reference in the numbering of nucleotide and amino acid positions. Molecular studies will be performed only for research purposes and will have no impact on the clinical management of study patients.

#### HIV and syphilis screening

HIV and Syphilis will be measured according to the Uganda National Guidelines [19]

#### Blood glucose

Blood glucose will be measured by Glucometer “Accu-Chek Active”, Narang Medical LTD.

#### Hemoglobin levels

Blood haemoglobin levels will be measured by Hemoglobin Testing System “Mission Ultra Hb”, Narang Medical LTD.

### Data analysis

For descriptive purposes continuous and ordinal variables data will be expressed as median with interquartile range. For categorical variables, percentages are calculated. Student’s t-test or analysis of variance (ANOVA) will be used to compare normally distributed numerical variables. Mann Whitney U-tests and Kruskal–Wallis tests will be used to compare numerical variables when normality cannot be assumed, while chi-squared tests will be used to compare categorical variables.

Association analysis will be carried out to identify risk factors for Plasmodium infection and adverse maternal or foetal outcomes. We will compare behavioural factors and adherence to IPTp (and type of IPTp regimen) to the incidence of symptomatic/severe malaria and adverse neonatal or foetal outcomes (miscarriage, stillbirth, low birthweight). Multivariable logistic regression models will be used to identify independent risk factors for the same clinical outcomes. A forward and backwards stepwise approach will used to include variables into the models, with a limit of P < 0.2.

A P-value of < 0.05 will be considered statistically significant. Final analyses will be conducted after the end of patient recruitment while interim analyses are planned at half 7 months from the incipit. Statistical analysis will be performed with R-software (R Foundation for Statistical Computing, Vienna, Austria).

## Discussion

According to the 2021 Essential Maternal and Newborn Clinical Care Guidelines for Uganda, for a woman with a normally progressing pregnancy the standard recommendation is a minimum of eight antenatal visits [26].

- Contact 1: Anytime ≤ 12 weeks.
- Contact 2: 13–20 weeks of gestation.
- Contact 3: 21–28 weeks of gestation.
- Contact 4: 30 weeks of gestation.
- Contact 5: 34 weeks of gestation.
- Contact 6: 36 weeks of gestation.
- Contact 7: 38 weeks of gestation.
- Contact 8: 40 weeks of gestation.

In addition, participants will be instructed to come to the clinic every time they are ill and will be evaluated at this point too. Outcomes will be assessed at the delivery or/and at the discharge if admitted to the hospital for any other causes related with the pregnancy or malaria (Table 1).

**Table 1:** Study time points

Time point	Study procedures and data collection
Recruitment (ANC visit)	– *Full questionnaire* administered by a trained healthcare worker to collect information on demographic (eg. age, area of residence), socioeconomic factors (eg. education, occupation), number of previous pregnancies,, bed net ownership and use, adherence to malaria chemoprevention, barriers to administration of IPTp, use of ITNs, risk perception of COVID-19 and Malaria will be administered – *Clinical examination* assessing the general wellbeing and nutritional status of the woman, along with routine measurements (including weight, height, auscultation, blood pressure and temperature). Gestational age will be assessed, when available, by obstretic ultrasound and, if unavailable, by pelvis examination performed by experienced midwives – *Collection of blood sample* that will be analyzed as follows: oMalaria diagnostic test with microscopy and, when positive, parasite count. Positive samples will be sent to the ISS. Molecular diagnosis will be performed to confirm microscopy results and to discriminate between *Plasmodium* species. *Plasmodium falciparum* positive samples will be analyzed for detection of single nucleotide polymorphisms in *P. falciparum* genes associated with artemisinin and SP resistance. Women diagnosed with malaria will be treated and followed according to Uganda Clinical Guidelines o HIV diagnostic test o Hemoglobin levels o Blood glucose levels o Syphilis testing (RPR) All women found to be HIV + at study entry will be referred for further evaluation and treatment
ANC visits	– Physical examination – Tests to be performed as per study procedure: – HB estimation at first contact and at 26 weeks with every pregnant woman: – HIV testing: first contact and 36w contact – Malaria screening: each ANC visit (maximum 8 times). Malaria diagnostic test with microscopy and, when positive, parasite count. Positive samples will be sent to the ISS. Molecular diagnosis will be performed to confirm microscopy results and to discriminate between *Plasmodium* species. *Plasmodium falciparum* Positive samples will be analyzed for detection of single nucleotide polymorphisms in P. falciparum genes associated with artemisinin and SP resistance. Women diagnosed with malaria will be treated and followed according to Uganda Clinical Guidelines
Any spontaneous visits to the hospital related with pregnancy and/or malaria	– Standardized history – Physical exam including temperature, pulse, and blood pressure measurement – Patients who are febrile (tympanic temperature > 38.0˚C) or report history of fever in the past 24 h will have blood obtained by finger prick for a thick blood smear. If the thick blood smear is positive, the patient will be diagnosed with malaria. If the thick blood smear is negative, the patient will be managed by study physicians for a non-malarial febrile illness. If the patient is afebrile and does not report a recent fever, a thick blood smear will not be obtained, except when following routine testing schedules – In patients with positive microscopy for *P. falciparum*, the first, pre-therapy blood sample collected as DBS will be sent for molecular diagnosis confirmation and for genetic analysis of antimalarial resistance to ISS. Furthermore, for hospitalized patients, parasitemia will be reassessed at day 3, as per WHO protocol [27] – Recruitment of the subpopulation of non-pregnant individuals will be undertaken at this time point
Delivery	– *Delivery information*: study staff will document details of the delivery, including date and time, type of delivery, estimated blood loss and any maternal, obstetrical or neonatal complications – *Fetal outcomes:* stillbirths, low birth weight, preterm birth – *Infant information:* Apgar score and birth weight with calibrated scales. At the time of delivery, women will undergo repeat rapid HIV testing based on national guidelines. If women are found to have become HIV-infected during pregnancy, both the mother and their newborn will be referred for care following local prevention of mother-to-child transmission guidelines [26] – Analyses of the Placenta Blood by microscopy to detect placental parasitemia

Data will be collected using semi-structured questioners and managed using REDCap electronic data capture tools hosted at “Catholic University of the Sacred Heart”, Rome, Italy [28], and will be recorded on standard study data collection forms and will be reviewed for accuracy and completion. Upon resolution of data forms errors/missing values, the form will be ready for data entry. The obtained results will be entered into a database. A database will be developed to accommodate data entry and management of the study's data. The database will be created with a standard data management software package, such as Microsoft Office. A file for each study form will be created.

### Ethical considerations

Pregnant women will be asked for written informed consent to participation to the study. In line with the Ugandan National Guidelines for Research involving Humans as Research Participants [29], women below the age of 18 will be considered emancipated minors. Every clinical outcome will be managed as per National Clinical Guidelines for the care of pregnant mothers. Counselling and related clinical support will be offered to participants who get pregnancy losses. This study will not introduce any clinical management strategies outside the national clinical guidelines.

We shall administer consent at two levels; first we shall administer and obtain consent for participation in the study and publication of future results and thereafter consent for collection and transportation of blood samples to Italy. The blood samples collected will be used only for the purpose of this study and will not be stored beyond the current study project. All unprocessed blood samples will be destroyed from the department of infectious disease, Italian National Institute of Health as biological sanitary waste, at the end of the project. The same standard of care will be guaranteed to all individuals irrespectively of participation. The dignity of study participants will be guaranteed by the investigators, as well as data confidentiality and the right to withdraw data and/or biological samples at any time. Consent to pregnant women and to mature and emancipated minors will be obtained according to Uganda Human Subjects Protection Guidelines [29].

For all malaria positive samples processed for molecular analysis in the present study, identifying information will be removed to provide appropriate protection of medical confidentiality and privacy. In this way, sensitive data cannot be linked or re-linked with identifiable human subjects, making anonymous each sample processed.

The polymorphisms analysis of the propeller domain of the *Pfk13, Pfdhfr* and *Pfdhps* genes will be performed by advanced amplifications techniques and subsequent Sanger sequencing in a highly specialized scientific laboratory. The blood samples collected in this study will be sent to a laboratory in the Italian National Institute of Health, Italy, in order to have access to the latest gene amplification techniques. To enhance local capacity building, one laboratory staff from Aber Hospital will be supported to attend a two weeks exposure at the reference laboratory in Italy.

The present study has been approved by the Lacor Hospital Institutional Research and Ethics Committee (prot. no LACOR-2022-95).

### Dissemination of results

Results from this research will be disseminated at various fora, including local and international scientific conferences, the Ministry of Health, technical working groups and meetings at district offices in Lango region involving participation of local leaders (Cultural, technical, and Political leaders). To better reach enrolled communities, the findings of the study will also be disseminated in places of worship and community health service points using the community outreaches being organized by CUAMM and the district local governments in Oyam and Kole districts. Findings will also be published in a peer reviewed journal.

## Data Availability

All data that will be generated or analysed during this study will be either included in the published article (and its supplementary information files) or will be made freely available to scientists wishing to use them for non-commercial purposes, without breaching participant confidentiality.

## Abbreviations

ACT: Artemisinin combination therapy
ANC: Antenatal care
CI: Confidence interval
COVID-19: Corona Virus Disease 2019
HIV: Human Immunodeficiency Virus
IPTp: Intermittent preventive treatment in pregnancy
LMICs: Low- and middle-income countries
MOH: Ministry of Health
NGO: Non-Governmental Organization
PNFP: Private not for profit
Pfdhfr: P. falciparum dihydrofolate reductase
Pfdhps: P. falciparum dihydropteroate synthase
Pfk13: P. falciparum kelch 13
RDT: Rapid diagnostic test
SP: Sulfadoxine-pyrimetamine
UDHS: Uganda Demographic and Health Survey
UNCST: Uganda national council of science and technology
WHO: World Health Organization

## References

[1] World Health Organization (2021). World malaria report 2021.

[2] Uganda—Malaria Indicator Survey 2018–2019. https://microdata.worldbank.org/index.php/catalog/3700. Accessed 6 Jun 2022.

[3] Namuganga JF, Briggs J, Roh ME, Okiring J, Kisambira Y, Sserwanga A (2021). Impact of COVID-19 on routine malaria indicators in rural Uganda: an interrupted time series analysis. Malar J.

[4] Desai M, Hill J, Fernandes S, Walker P, Pell C, Gutman J (2018). Prevention of malaria in pregnancy. Lancet Infect Dis.

[5] D’Alessandro U, Hill J, Tarning J, Pell C, Webster J, Gutman J (2018). Treatment of uncomplicated and severe malaria during pregnancy. Lancet Infect Dis.

[6] Rogerson SJ, Desai M, Mayor A, Sicuri E, Taylor SM, van Eijk AM (2018). Burden, pathology, and costs of malaria in pregnancy: new developments for an old problem. Lancet Infect Dis.

[7] Cates JE, Unger HW, Briand V, Fievet N, Valea I, Tinto H (2017). Malaria, malnutrition, and birthweight: A meta-analysis using individual participant data. PLoS Med.

[8] Kwenti TE (2018). Malaria and HIV coinfection in sub-Saharan Africa: prevalence, impact, and treatment strategies. Res Rep Trop Med.

[9] WHO Guidelines for malaria. https://www.who.int/publications-detail-redirect/guidelines-for-malaria. Accessed 10 Nov 2021.

[10] Desai M, Gutman J, Taylor SM, Wiegand RE, Khairallah C, Kayentao K (2016). Impact of sulfadoxine-pyrimethamine resistance on effectiveness of intermittent preventive therapy for malaria in pregnancy at clearing infections and preventing low birth weight. Clin Infect Dis Off Publ Infect Dis Soc Am.

[11] van Eijk AM, Hill J, Larsen DA, Webster J, Steketee RW, Eisele TP (2013). Coverage of intermittent preventive treatment and insecticide-treated nets for the control of malaria during pregnancy in sub-Saharan Africa: a synthesis and meta-analysis of national survey data, 2009–11. Lancet Infect Dis.

[12] Olaleye A, Okusanya BO, Oduwole O, Esu E, Meremikwu M (2019). A systematic review and meta-analysis of dihydroartemisinin-piperaquine versus sulphadoxine-pyrimethamine for malaria prevention in pregnancy. Int J Gynaecol Obstet.

[13] Thomson R, Beshir KB, Cunningham J, Baiden F, Bharmal J, Bruxvoort KJ (2019). pfhrp2 and pfhrp3 Gene deletions that affect malaria rapid diagnostic tests for plasmodium falciparum: analysis of archived blood samples from 3 African Countries. J Infect Dis.

[14] Okell LC, Ghani AC, Lyons E, Drakeley CJ (2009). Submicroscopic infection in Plasmodium falciparum-endemic populations: a systematic review and meta-analysis. J Infect Dis.

[15] Rogerson SJ, Hviid L, Duffy PE, Leke RF, Taylor DW (2007). Malaria in pregnancy: pathogenesis and immunity. Lancet Infect Dis.

[16] Balikagala B, Fukuda N, Ikeda M, Katuro OT, Tachibana S-I, Yamauchi M (2021). Evidence of Artemisinin-resistant Malaria in Africa. N Engl J Med.

[17] Blasco B, Leroy D, Fidock DA (2017). Antimalarial drug resistance: linking *Plasmodium falciparum* parasite biology to the clinic. Nat Med.

[18] Mbonye AK, Birungi J, Yanow SK, Shokoples S, Malamba S, Alifrangis M (2015). Prevalence of *Plasmodium falciparum* resistance markers to sulfadoxine-pyrimethamine among pregnant women receiving intermittent preventive treatment for malaria in Uganda. Antimicrob Agents Chemother.

[19] Uganda Clinical Guidelines 2016 | Ministry of Health Knowledge Management Portal. http://library.health.go.ug/publications/guidelines/uganda-clinical-guidelines-2016. Accessed 12 Nov 2021.

[20] Bater J, Lauer JM, Ghosh S, Webb P, Agaba E, Bashaasha B (2020). Predictors of low birth weight and preterm birth in rural Uganda: findings from a birth cohort study. PLoS ONE.

[21] De Beaudrap P, Turyakira E, White LJ, Nabasumba C, Tumwebaze B, Muehlenbachs A (2013). Impact of malaria during pregnancy on pregnancy outcomes in a Ugandan prospectivecohort with intensive malaria screening and prompt treatment. Malar J.

[22] Metcalfe C. Biostatistics: a foundation for analysis in the health sciences. 7th edn. Wayne W. Daniel, Wiley, 1999. No. of. pages: xiv+755+appendices. Price: £28.95. ISBN 0-471-16386-4. Stat Med. 2001;20:324–6.

[23] Snounou G, Viriyakosol S, Zhu XP, Jarra W, Pinheiro L, do Rosario VE (1993). High sensitivity of detection of human malaria parasites by the use of nested polymerase chain reaction. Mol Biochem Parasitol..

[24] Taylor SM, Parobek CM, DeConti DK, Kayentao K, Coulibaly SO, Greenwood BM (2015). Absence of putative artemisinin resistance mutations among *Plasmodium falciparum* in Sub-Saharan Africa: a molecular epidemiologic study. J Infect Dis.

[25] Menegon M, Pearce RJ, Inojosa WO, Pisani V, Abel PM, Matondo A (2009). Monitoring for multidrug-resistant *Plasmodium falciparum* isolates and analysis of pyrimethamine resistance evolution in Uige province, Angola. Trop Med Int Health.

[26] Ministry of Health. Essential Maternal and Newborn Clinical Care Guidelines For Uganda | Ministry of Health Knowledge Management Portal.

[27] WHO Guidelines for malaria—18 February 2022. 2022;240.

[28] Harris PA, Taylor R, Minor BL, Elliott V, Fernandez M, O’Neal L (2019). The REDCap consortium: Building an international community of software platform partners. J Biomed Inform.

[29] Uganda National Council for Science and Technology (UNCST). National Guidelines for Research involving Humans as Research Participants. 2014.

